# Efficacy of opioid-free anesthesia in short-term recovery following laparoscopic-assisted colorectal tumor resection: a randomized trial

**DOI:** 10.3389/fonc.2025.1588623

**Published:** 2025-07-02

**Authors:** Liang Zhang, Xian-Hua Yu, Hui-Ming Zhang, Sheng Wang, Jian-Long Chen, Xue-Shan Li, Zhi-Yuan Chen

**Affiliations:** ^1^ Department of Anesthesiology, The Second Affiliated Hospital of Fujian Medical University, Quanzhou, China; ^2^ Department of Anesthesiology, Sanming First Hospital Affiliated to Fujian Medical University, Sanming, China; ^3^ Department of Pain, Sanming First Hospital Affiliated to Fujian Medical University, Sanming, China

**Keywords:** colorectal tumor, laparoscopic surgery, opioid-free anesthesia, thoracic epidural anesthesia, transversalis fascia nerve block

## Abstract

**Objective:**

This study aims to evaluate whether opioid-free anesthesia is non-inferior to opioid-based anesthesia in terms of short-term recovery quality in patients undergoing laparoscopic-assisted colorectal tumor resection.

**Methods:**

A randomized controlled trial was conducted with 102 participants, who were randomly assigned to one of two groups: opioid-free general anesthesia with thoracic epidural anesthesia (OFA) group and opioid-based general anesthesia with compound transversalis fascia nerve block (OA) group. The primary observation outcomes were the preoperative and postoperative Quality of Recovery-40 (QoR-40) questionnaire scores.

**Results:**

No statistically significant differences were observed in preoperative or postoperative QoR-40 scores between the two groups (*p* = 0.05). However, the OFA group demonstrated a significantly longer recovery time in the recovery room compared to the OA group (*p*< 0.05). No significant differences were observed between the two groups in postoperative nausea and vomiting, time to first meal after surgery, postoperative drainage tube removal time, postoperative sufentanil dose, or postoperative 24-hour numerical rating scale (*p* > 0.05).

**Conclusion:**

Opioid-free general anesthesia is not superior to opioid-based general anesthesia with transversalis fascia nerve block in terms of short-term postoperative recovery quality following laparoscopic-assisted colorectal tumor resection.

**Clinical Trial Registration:**

https://www.chictr.org.cn/, identifier 2023-12-08.

## 1 Introduction

Opioid-free anesthesia (OFA) is a modern anesthetic approach that utilizes a combination of non-opioid drugs and/or techniques as part of general anesthesia. It has been shown to be effective in surgeries for special populations, including patients with chronic obstructive pulmonary disease, obesity, acute or chronic opioid addiction, sleep apnea, and those undergoing cancer surgery (1, 2). The primary advantage of OFA is its ability to reduce or eliminate opioid use, thereby minimizing opioid-related side effects such as nausea and vomiting, postoperative respiratory depression, and opioid-induced immunosuppression and cognitive dysfunction. Additionally, OFA has been associated with improved postoperative pain control, reduced postoperative pain sensitivity, decreased persistent postoperative pain, and a lower risk of opioid addiction and dependence (1, 3–6). This study aimed to evaluate whether opioid-free anesthesia is non-inferior to opioid-based anesthesia in terms of short-term recovery quality in patients who underwent laparoscopic-assisted colorectal tumor resection.

## 2 Data and methods

### 2.1 Ethics and research design

This randomized controlled trial was approved by the Ethics Committee of Sanming First Hospital Affiliated to Fujian Medical University, (approval number: (2023) No. 54), and was registered with the China Clinical Trial Registry (Registration website: https://www.chictr.org.cn/, Registration number: ChiCTR2300078462, Registration number:2023-12-08). Patients were enrolled after registration approval. This study was conducted in accordance with the Guidelines of the Declaration of Helsinki and the Harmonized Standards for Clinical Trial Reporting. Written informed consent was obtained from all enrolled patients.

### 2.2 Participants

**Figure 1:**
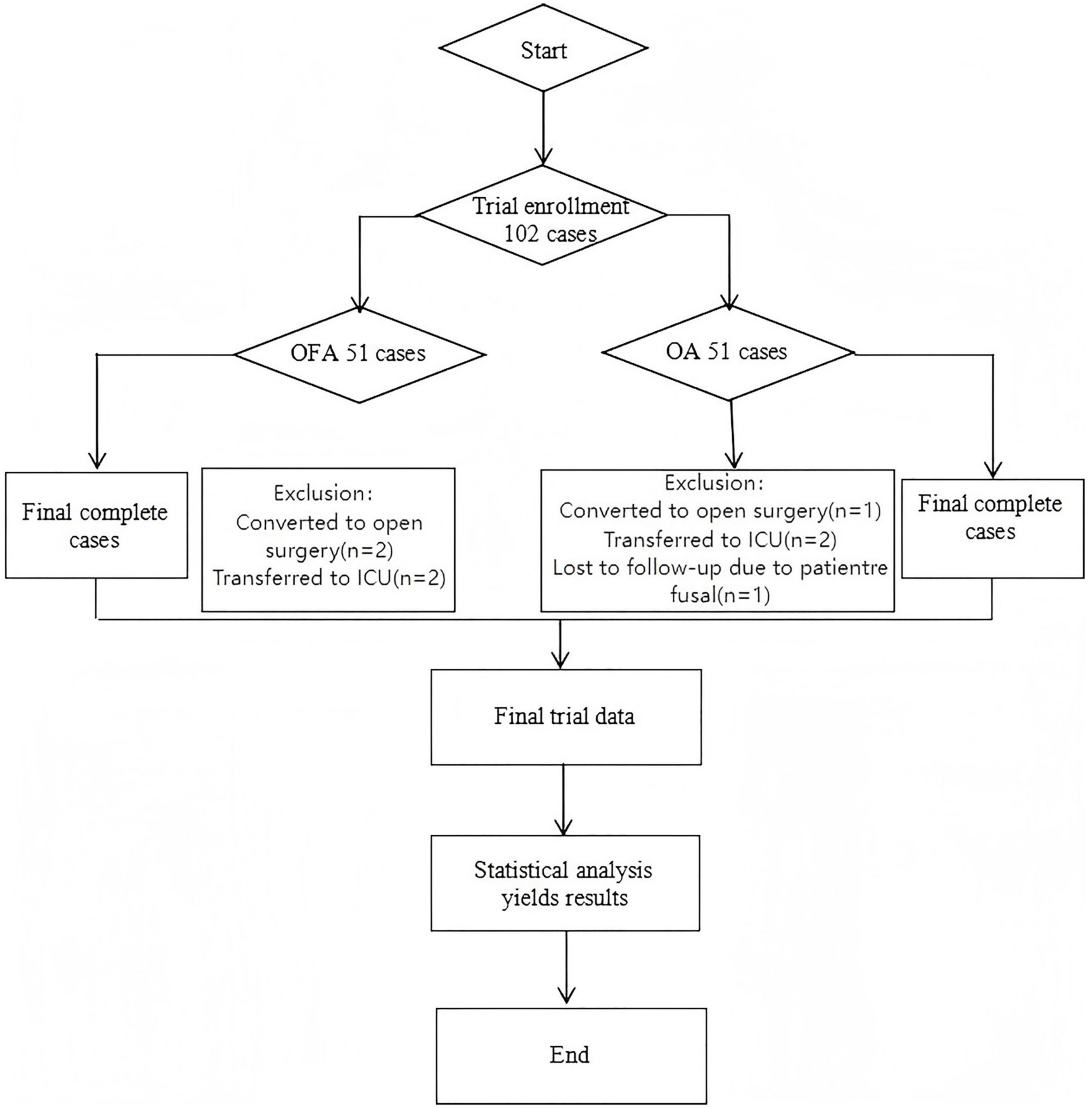
Flowchart.

Patients aged 18 to 80 years, irrespective of their gender, having an American Society of Anesthesiologists (ASA) grade I to III, a body mass index (BMI) between 18 and 30 kg/m², and scheduled to undergo laparoscopic-assisted colorectal tumor resection were enrolled in the study. Exclusion criteria: 1. Severe hepatic insufficiency (defined as a prothrombin ratio< 15), 2. Coagulation dysfunction, 3. Cardiac conduction system abnormalities (sinus, atrioventricular, or intraventricular block), 4. Cutaneous nerve block infection, 5. History of analgesic or psychotropic medication use due to chronic pain, hypersensitivity to local or general anesthetics, 6. Contraindication to neuraxial anesthesia, 7. Severe psychiatric disorder or communication difficulties, emergency surgery, or refusal to participate. The flowchart of patient inclusion is presented in **Figure 1**.

### 2.3 Anesthesia methods

After the patient entered the operating room, peripheral venous access was established, and the electrocardiogram, blood pressure, blood oxygen saturation, body temperature, and bispectral index system (BIS) were routinely monitored. Under local anesthesia, radial artery puncture and catheterization were performed, and invasive arterial pressure was monitored. A computer-generated random number table was used to develop the randomization protocol, and the assigned numbers were sealed in opaque envelopes, which were opened by individuals not involved in the study at the time of inclusion. Prior to anesthesia induction, both the anesthesiologists and surgeons were blinded to group allocation. During the surgical procedure, group assignment was revealed to the anesthesiologist and surgeon to ensure appropriate intraoperative management. However, the anesthesiologist was only responsible for collecting intraoperative data. Postoperative data, including those from the recovery room, were collected by independent personnel who remained blinded to group assignments. Furthermore, surgeons were instructed not to disclose group allocation to patients or their families after surgery to maintain the integrity of the blinding protocol. The study drug was prepared by staff members who were not involved in the study and was provided as an unidentified solution.

OFA group: Epidural puncture and catheterization were performed at T11–T12, with a local anesthetic solution containing 0.25% ropivacaine and 0.5% lidocaine administered into the epidural space. A test dose of 3 ml of 2% lidocaine was injected, and general anesthesia induction was initiated after confirming its correct placement. A loading dose of dexmedetomidine (1 μg/kg) was infused over 30 minutes before induction, followed by midazolam (0.05 mg/kg), lidocaine (1.5 mg/kg), propofol (2 mg/kg), and cisatracurium (0.2 mg/kg) for induction. After three minutes of preoxygenation, endotracheal intubation was performed using a laryngoscope, with the endotracheal tube lubricated with dyclonine. General anesthesia was maintained with sevoflurane, propofol, and dexmedetomidine (0.3 μg/kg/h) throughout the procedure. BIS levels were kept between 40 and 60 by adjusting sevoflurane and propofol dosages, while intraoperative muscle relaxation was achieved through intermittent intravenous cisatracurium administration. Epidural anesthesia was initiated with an initial 10 ml dose, followed by 5 ml every two hours until surgery was completed. Dexmedetomidine administration was stopped upon colorectal dissection.

Opioid anesthesia (OA) group: Patients underwent bilateral transversalis fascia nerve block with 40 ml of 0.25% ropivacaine after the monitoring device was connected. Once the block plane was confirmed, the patient received midazolam (0.05 mg/kg), sufentanil (0.3–0.5 μg/kg), propofol (2 mg/kg), and cisatracurium (0.2 mg/kg) for induction. After three minutes of preoxygenation, endotracheal intubation was performed using a laryngoscope, with the endotracheal tube lubricated with dyclonine. General anesthesia was maintained with sevoflurane and remifentanil throughout the procedure. BIS levels were kept between 40 and 60 by adjusting the dosages of sevoflurane and remifentanil, while intraoperative muscle relaxation was ensured through intermittent intravenous cisatracurium administration.

Volume-controlled mechanical ventilation was utilized during surgery, with ventilation parameters set as follows: tidal volume of 7 to 8 ml/kg, 50% inspired oxygen concentration, and an oxygen flow rate of 2 l/min. Respiratory parameters were adjusted to maintain end-tidal carbon dioxide between 30 and 45 mmHg. Based on baseline blood pressure measured upon entering the operating room, norepinephrine was administered to sustain the mean arterial pressure (MAP) within 20% of the baseline value or maintain MAP > 65 mmHg, while heart rates (HR) below 50 beats per minute were managed with atropine (0.5 mg). Intraoperative temperature management included infusion warming, maintaining the operating room temperature above 23°C, and using a heater to keep core temperature between 36 and 37°C. At the conclusion of the surgery, all medications were discontinued, muscle relaxants were reversed with atropine and neostigmine, and midazolam was antagonized with flumazenil. The endotracheal tube was removed once the patient responded to verbal stimuli, had stable circulation, exhibited a bucking or swallowing reflex, and achieved spontaneous breathing with a tidal volume exceeding 5 ml/kg. Following five minutes of observation, the patient was transferred to the recovery room when SpO_2_ remained above 95% and circulation was stable. All patients in this trial provided informed consent for postoperative analgesia. Upon returning to the ward and leaving the recovery room, patient-controlled intravenous analgesia (PCIA) was initiated after a loading dose. The analgesic pump formula consisted of 200 ml (sufentanil 200 μg + ondansetron 16 mg), with a continuous infusion rate of 2 ml/h, a single dose of 2 ml, and a lockout interval of 10 minutes.

### 2.4 Observation indicators

The preoperative and 24-hour postoperative Quality of Recovery-40 (QoR-40) questionnaire score, general patient data, and hemodynamic parameters at key intraoperative time points were recorded. Additionally, postoperative nausea and vomiting (PONV), the time of postoperative drainage tube removal, postoperative feeding initiation, postoperative length of stay (LOS), the total sufentanil dosage used for 24-hour postoperative analgesia, and the 24-hour Numeric Rating Scale (NRS) for pain assessment were evaluated.

### 2.5 Sample size calculation and statistical analysis

This study is a randomized controlled trial with two groups: the OFA group and the OA group. In the preliminary experiment, the mean QoR-40 score for 10 patients in each group was 185.18 points in the OFA group and 181.35 points in the OA group. Based on a literature review, a difference of 6.3 points was considered the minimal clinically significant difference (7). A one-sided test was conducted with α = 0.05, and statistical power was set at 1 – β = 0.9. Using the Medical Statistical Assistant V11.8 software, the sample size for both the OFA and OA groups was calculated to be 44 cases each. To account for a potential 15% loss to follow-up or refusal to participate in follow-up, additional participants were included. Thus, the final sample size consisted of 102 participants, with 51 in each group.

Data analysis was performed using SPSS 27.0 statistical software. Quantitative variables were first tested for normality. Depending on the distribution, either the independent samples t-test or the Mann–Whitney U-test was applied for analysis. These variables included the QoR-40 score, age, operation time, and anesthesia time, and were expressed as mean ± standard deviation. Qualitative variables, such as sex and PONV, were analyzed using the Chi-square test or Fisher’s exact test. A *p*-value of< 0.05 was considered statistically significant.

## 3 Results

### 3.1 Enrollment

A total of 102 patients who underwent laparoscopic-assisted colorectal resection were enrolled in the study based on the inclusion criteria and were randomly assigned to the OFA group (n = 51) or the OA group (n = 51). During the perioperative period, three patients from the OFA group were excluded: one was converted to open surgery, and two were admitted to the intensive care unit (ICU) postoperatively. Similarly, three patients from the OA group were excluded, all of whom required ICU admission after surgery. Ultimately, 96 patients completed the study and were included in the statistical analysis, with 48 patients in the OFA group and 48 in the OA group.

### 3.2 General data of the patients

There were no significant differences between the two groups in general characteristics, including sex (male/female), age (years), height (cm), weight (kg), BMI, anesthesia duration, and operation time (*p* > 0.05) (**Table 1**).

**Table 1:** Comparison of general data ( $\bar{x}$ ± *s*).

Items	OFA group (n = 48)	OA group (n = 48)	*p-*value
Sex (male/female)	26/22	28/20	P=0.68
Age (years)	62.96 ± 8.84	64.3 ± 10.55	P=0.47
Height (cm)	160.25 ± 7.48	161 ± 6.35	P=0.27
Weight (kg)	56.71 ± 10.49	58.69 ± 8.40	P=0.31
Body mass index (kg/m^2^)	21.97 ± 3.09	22.39 ± 2.83	P=0.493
Anesthesia time (min)	302.71 ± 48.26	300 ± 63.36	P=0.828
Operation time (min)	222. 19 ± 43.73	222.08 ± 65.07	P=0.892

### 3.3 Comparison of hemodynamics and intraoperative norepinephrine doses (secondary observation indicators)

In terms of hemodynamics: 1. A statistically significant difference in HR was observed between the two groups at the time of skin incision and 5 minutes after tracheal tube removal (*p*< 0.05). Specifically, HR was higher in the OFA group than in the OA group at the time of skin incision, whereas HR was higher in the OA group than in the OFA group 5 minutes after tracheal tube removal. 2. A statistically significant difference in MAP was found between the two groups at 1 minute after tracheal intubation, during skin suturing, and 5 minutes after tracheal tube removal (*p*< 0.05). The OFA group had a higher MAP than the OA group at 1 minute after tracheal intubation, as well as during skin suturing. However, at 5 minutes after tracheal tube removal, the MAP of the OA group was higher than that of the OFA group. 3. No significant difference in HR was noted between the two groups at the time of ward admission, entry into the operating room, 1 minute after tracheal intubation, 5 minutes after pneumoperitoneum, and during skin suturing (*p* > 0.05). 4. No significant difference in MAP was found between the two groups at ward admission, entry into the operating room, skin incision, and 5 minutes after pneumoperitoneum (*p* > 0.05). Additionally, there was no significant difference between the two groups in intraoperative norepinephrine demand or norepinephrine demand per unit of anesthesia time (*p* > 0.05) (**Table 2**).

**Table 2:** Comparison of MAP and HR between the two groups ( $\bar{x}$ ± *s*).

Time points	OFA group (n = 48)	OA group (n = 48)	*p-*value
HR in the ward (beats/minute)	77.19 ± 9.13	76.40 ± 10.01	P=0.428
HR upon operating room admission (beats/minute)	76.38 ± 10.25	76.29 ± 11.67	P=0.898
HR after tracheal intubation (beats/minute)	72.73 ± 11.28	71.54 ± 14.16	P=0.375
HR at skin incision (beats/minute)	63.77 ± 11.09	57.98 ± 9.39	P=0.007
HR at 5 min after pneumoperitoneum (beats/minute)	64.88 ± 12. 14	60.57 ± 10.43	P=0.077
HR at skin suture (beats/minute)	64.92 ± 8.99	63.60 ± 11.43	P=0.215
HR after tracheal tube removal (beats/minute)	76.83 ± 11.79	82.60 ± 13.56	P=0.028
MAP in the ward (mmHg)	97.24 ± 8.61	98.16 ± 10.71	P=0.950
MAP upon operating room admission (mmHg)	102.46 ± 11.33	102.60 ± 12.17	P=0.950
MAP after tracheal intubation (mmHg)	98.42 ± 11.66	92.19 ± 17.35	P=0.050
MAP at skin incision (mmHg)	83.12 ± 11.44	84.15 ± 13.96	P=0.881
MAP at 5 min after pneumoperitoneum (mmHg)	94.51 ± 11.13	91.08 ± 14.25	P=0.192
MAP at skin suture (mmHg)	85.24 ± 8.41	80.78 ± 8.76	P=0.008
MAP after tracheal tube removal (mmHg)	97.90 ± 14.10	104.68 ± 12.67	P=0.015
Norepinephrine dose (µg)	460.25 ± 454.10	574.17 ± 512.85	P=0.207
Norepinephrine dose per anesthesia time unit (µg/min)	1.52 ± 1.49	1.97 ± 1.82	P=0.174

### 3.4 Main observation indicators: preoperative and 24-hour postoperative QoR-40 score

There was no statistically significant difference in the preoperative QoR-40 score between the two groups (*p* > 0.05). Similarly, no statistically significant difference was observed in the QoR-40 score between the two groups at 24 hours postoperatively (*p* = 0.05) (**Table 3**).

**Table 3:** QoR-40 score before and 24 hours after surgery ( $\bar{x}$ ± *s*).

Items	OFA group (n = 48)	OA group (n = 48)	*p-*value
Before surgery	199.50 ± 0.72	199.23 ± 1.51	P=0.979
24 hours after surgery	184.85 ± 3.04	183.81 ± 3.13	P=0.05

### 3.5 Secondary observation indicators

A statistically significant difference was observed in the wake-up time in the recovery room, with patients in the OFA group exhibiting a longer wake-up time than those in the OA group. There was no significant difference between the two groups in terms of PONV, time to first meal post-surgery (in days), or postoperative drainage tube removal time (in days) (*p* > 0.05). Additionally, no significant difference was found between the groups in postoperative sufentanil dosage (in µg) or 24-hour NRS after surgery (*p* > 0.05) (**Table 4**).

**Table 4:** Comparison of postoperative observation indicators ( $\bar{x}$ ± *s*).

Postoperative observation indicators	OFA group (n = 48)	OA group (n = 48)	*p-*value
PONV (yes/no)	10/38	13/35	P=0.470
Postoperative feeding time (days)	4.9 ± 1.46	5.19 ± 1.68	P=0.450
Postoperative drainage tube removal time (days)	7.85 ± 1.62	8.21 ± 1.76	P=0.147
Postoperative length of stay (days)	10. 13 ± 2.13	10.30 ± 2.67	P=0.848
Sufentanil dose (µg)	72.96 ± 14.85	71.73 ± 13.03	P=0.714
NRS	2.73 ± 0.61	2.63 ± 0.73	P=0.171
Wake-up time in the recovery room (min)	49.31 ± 22.75	43.33 ± 27.64	P=0.009

## 4 Discussion

This randomized controlled trial evaluated the effects of OFA using non-opioid techniques of thoracic epidural anesthesia and opioid general anesthesia with compound transversalis fascia nerve block in patients undergoing laparoscopic-assisted colorectal tumor resection, focusing on short-term quality of recovery. The findings indicate that there is no statistically significant difference in quality of recovery between patients receiving OFA with thoracic epidural anesthesia and those receiving OA with compound transversalis fascia nerve block. Patients who underwent OFA experience a longer wake-up time in the post-anesthesia care unit compared to those in the OA group. No significant differences were observed between the two groups regarding PONV, time to first meal after surgery, postoperative drainage tube removal time, or postoperative LOS.

When comparing the short-term quality of recovery between patients receiving OFA with epidural nerve block during the perioperative period and those undergoing low-OA with compound transversalis fascia nerve block under the enhanced recovery after surgery protocol, OFA does not demonstrate superiority. Non-opioid techniques, including neuraxial anesthesia and precise regional nerve blocks, were implemented to minimize opioid use (8). The QoR-40 score was used to assess the quality of patient recovery, as it serves as a global measure incorporating five dimensions of health: physical comfort, emotional state, physical independence, psychological support, and pain. Generally, a 10-point difference in the QoR-40 score corresponds to a 15% improvement in recovery quality. The efficacy and reliability of the QoR-40 have been validated in prior studies, and it has been widely utilized to evaluate postoperative recovery across different anesthesia and surgical techniques (9).

Furthermore, the Perioperative Medicine Standardized Endpoint Program recommends QoR-40 as a standardized measure of patient comfort (10, 11). Based on previous research evaluating OFA’s impact on early postoperative recovery following major surgery, patients receiving OFA scored 6.2 points higher at 24 hours (Quality of Recovery-15 scale) than those receiving OA (12). In this study, patients in the OFA group scored only 1 point higher than those in the OA group, aligning with findings by Myles et al. However, the wide variation in scores suggests that preoperative transversalis fascia nerve block in patients with OA may have contributed to the outcome. This technique can reduce intraoperative pain stimuli, and since all patients underwent laparoscopic surgery, which is renowned for its advantages of minimal intraoperative stimulation and reduced postoperative pain—both groups required lower opioid doses perioperatively.

Consequently, the reduced opioid consumption in patients with OA likely mitigated opioid-related adverse effects. As observed in the results, while the QoR-40 score was slightly higher in the OFA group, the difference between groups was not statistically significant. This may also explain the absence of significant differences in PONV, time to first meal after surgery, postoperative drainage tube removal time, and LOS between the two groups.

The findings of this trial also indicate that patients in the OFA group had a longer wake-up time in the recovery room, which may be attributed to the perioperative administration of dexmedetomidine. In contrast, the OA group received sufentanil and ultra-short-acting remifentanil for analgesia, sevoflurane for sedation, and cisatracurium for muscle relaxation maintenance. The half-lives of these drugs are relatively short, and both remifentanil and sevoflurane, which were maintained throughout surgery, undergo rapid metabolism, facilitating quicker patient recovery. However, in the OFA group, dexmedetomidine was used and maintained until intestinal anastomosis, which likely contributed to the delayed wake-up time. Previous studies have reported that dexmedetomidine administration, both in healthy volunteers and critically ill patients, leads to sedation and delayed emergence from general anesthesia due to its pharmacokinetics (13–23). These findings align with the results, as patients in the OFA group exhibited longer wake-up times following dexmedetomidine administration. Following endotracheal tube removal, HR and MAP were higher in the OA group compared to the OFA group. These results indicate that patients in the OFA group demonstrated greater tolerance to stimulation and enhanced hemodynamic stability during extubation. This is consistent with the findings of Hontoir and Goettel et al., who reported that OFA is associated with less respiratory depression and greater hemodynamic stability during the wake-up period compared to OA (24, 25).

This study has several limitations. First, patients with critical illness were excluded during the trial, which limits the generalizability of the findings. Therefore, the safety and applicability of the results require further validation. Second, this was a single-center, small-sample clinical trial, which may limit its persuasiveness. A multi-center, large-sample trial is necessary to enhance the robustness and reliability of the findings. It is worth noting that the recovery room arousal time was significantly longer in the OFA group compared to the OA group. This prolonged recovery time may have practical implications for resource utilization and patient satisfaction, and warrants further investigation in future studies. Finally, this study did not investigate the effects on tumor immune function, long-term recovery, or tumor recurrence follow-up, which will be explored in future research.

## 5 Conclusion

Opioid-free general anesthesia is not superior to opioid general anesthesia in terms of short-term postoperative recovery quality in patients undergoing laparoscopic-assisted colorectal tumor resection.

## Data Availability

The raw data supporting the conclusions of this article will be made available by the authors, without undue reservation.
